# Lactylation in colorectal cancer: Unveiling novel mechanisms in metabolism, progression and therapeutic targeting

**DOI:** 10.1002/ctm2.70629

**Published:** 2026-02-19

**Authors:** Ming Liu, Weiwei Li, Yi Ji, Yanqing Chen, Guoli Wei, Jiege Huo, Tao Gui

**Affiliations:** ^1^ Affiliated Hospital of Integrated Traditional Chinese and Western Medicine Nanjing University of Chinese Medicine Nanjing China; ^2^ The Third Clinical Medical College Nanjing University of Chinese Medicine Nanjing China; ^3^ Jiangsu Province Academy of Traditional Chinese Medicine Nanjing China

**Keywords:** chemoresistance, colorectal cancer, gut microbiota, lactylation, tumour microenvironment

## Abstract

**Background:**

Colorectal cancer is a leading cause of cancer mortality characterised by a unique metabolic microenvironment and complex interactions with the gut microbiota. Lactylation, a novel post‐translational modification derived from lactate, has emerged as a key epigenetic regulator connecting metabolic reprogramming to gene expression. While its general roles in cancer are recognised, the tissue‐specific regulatory network of lactylation in colorectal cancer—particularly its interplay with the gut microbiome and specific chemotherapy resistance mechanisms—remains underexplored.

**Main body:**

This review systematically dissects the dynamic ‘writer‒eraser‒reader’ network of lactylation, highlighting its distinct oncogenic functions in colorectal cancer. We reveal a critical ‘microbiome‒metabolism‒epigenetics’ axis in which gut flora‐derived metabolites (including D‐lactate) remodel the tumour microenvironment and drive immune evasion. Beyond histone modifications, we emphasise the pivotal role of non‐histone lactylation targets (e.g., eEF1A2, PD‐L1) in orchestrating malignant proliferation and promoting liver metastasis by priming the pre‐metastatic niche. Furthermore, we elucidate novel mechanisms by which lactylation induces resistance to standard chemotherapeutic agents (5‐fluorouracil and oxaliplatin), specifically through the enhancement of DNA repair and the suppression of ferroptosis. We also critically evaluate the pharmacological challenges hindering clinical translation, such as the poor selectivity of current broad‐spectrum inhibitors.

**Short conclusion:**

Lactylation serves as a fundamental metabolic‒epigenetic link driving aggressive phenotypes in colorectal cancer. By delineating these tissue‐specific mechanisms and proposing next‐generation site‐specific targeting strategies, this review provides a theoretical foundation for developing precision medicine interventions to overcome therapy resistance in colorectal cancer patients.

## Keypoints/Highlights

1. This review is the first to summarise the role of lactylation in colorectal cancer (CRC) pathogenesis comprehensively.
2. We discover that lactylation acts as a crucial metabolic sensor linking glycolysis to CRC malignant behaviours.
3. Our study finds that lactylation promotes CRC immune evasion by modulating the tumour microenvironment.
4. We demonstrate that lactylation contributes significantly to chemoresistance in CRC cells.
5. This work provides a new theoretical basis for targeting lactate metabolism and lactylation in CRC therapy.

## Graphical abstract

Lactate accumulation links metabolic reprogramming to epigenetic regulation via protein lactylation. Lactylation drives colorectal cancer proliferation, metastasis, immune escape and chemoresistance. Microbiota‐derived lactate shapes a tumour‐specific lactylation landscape in colorectal cancer. Targeting lactylation pathways offers new opportunities for precision cancer therapy.

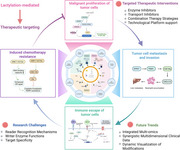

## 1 INTRODUCTION

Colorectal cancer (CRC) is a malignant tumour arising in the colon and rectum. It is the third most commonly diagnosed cancer and the second leading cause of cancer‐related death worldwide, posing a major threat to global health.^1^, ^2^ In 2020, more than 1.9 million new cases and approximately 930 000 deaths were reported globally, and these numbers are projected to rise to 3.2 million cases and 1.6 million deaths by 2040, particularly in countries with high or very high human development index.^3^ Although the incidence of CRC among middle‐aged and older adults has declined in some high‐income countries due to screening and lifestyle changes,^4^ it is increasing among younger populations. Moreover, many transitioning countries continue to experience rising incidence rates, likely driven by westernised diets and the growing prevalence of overweight and obesity. Because early‐stage CRC often presents with nonspecific or no symptoms, many patients are diagnosed at advanced stages, when prognosis is poor. Current treatments for CRC include surgery, chemotherapy, radiotherapy and targeted therapy, but their efficacy remains limited in advanced disease. Although early screening methods, such as colonoscopy and faecal occult blood testing, together with the removal of precancerous lesions, can significantly reduce CRC incidence and mortality, many patients miss these opportunities due to the lack of early symptoms. In addition, CRC frequently metastasises to distant organs, particularly the liver and lungs, further complicating treatment.^5^, ^6^ Given the molecular heterogeneity of CRC, precision therapy remains challenging, underscoring the urgent need to identify new therapeutic targets and develop more effective treatment strategies.

Since its discovery in 1780, lactate has been regarded as a harmful metabolic byproduct of hypoxia.^7^ However, in 2019, Zhao and coworkers^8^ identified a novel post‐translational modification (PTM), termed lactylation (Kla), in which lactate derived from glycolysis forms a lactyl group that covalently binds to lysine residues on proteins. As the end product of glycolysis, lactate accumulates significantly in the tumour microenvironment (TME) and can act as a signalling molecule regulating multiple processes. Lactylation influences gene expression and cellular functions, linking metabolic states with epigenetic regulation. Notably, Kla occurs not only on histones but also on a wide range of non‐histone proteins.

Recent studies published in leading journals such as *Nature and Cell* have highlighted the role of lactate metabolism and lactylation in tumour immune regulation and therapy resistance. A 2023 *Cell* study showed that lactylation promotes immune evasion by regulating immune checkpoint expression, while a 2024 Nature report demonstrated that lactylation enhances DNA damage repair, thereby driving tumour resistance.^9^ These findings further underscore the central role of lactylation in cancer research. Moreover, lactate metabolism and lactylation have been shown to shape the tumour immune microenvironment and therapeutic resistance in multiple cancers, including breast cancer and hepatocellular carcinoma (HCC), indicating a broadly conserved mechanism.^9^ Notably, CRC, characterised by a strong reliance on glycolysis and substantial lactate accumulation, represents an especially suitable disease model for investigating lactylation‐mediated metabolic–epigenetic regulatory pathways.

In CRC, lactylation is increasingly recognised as a key regulator of tumour progression, metabolism and immune evasion. Although its roles in immune escape and drug resistance have been reported in cancers such as breast and liver cancer, the specific mechanisms of lactylation in CRC remain poorly defined. Most current studies focus on individual molecules or isolated pathways, lacking an integrated understanding of the ‘lactate–lactylation–function–target’ axis.^10^, ^11^, ^12^ In particular, it is unclear whether lactylation promotes immune evasion and chemoresistance in CRC by regulating immune checkpoint expression, DNA damage repair, and metabolic reprogramming. Therefore, systematic investigation of lactylation in CRC is urgently needed to bridge the gap between metabolism and epigenetic regulation and to support the development of novel therapeutic strategies to overcome resistance to immunotherapy and chemotherapy.

Although several recent reviews have discussed lactylation across multiple cancer types, a systematic and mechanism‐focused synthesis centred on CRC remains lacking. This gap is particularly important because CRC is a metabolically active tumour with a highly complex microenvironment. Previous studies have rarely examined lactylation from an integrated perspective that considers tissue specificity, microenvironmental interactions and therapeutic response. Compared with other malignancies, CRC displays unique anatomical and metabolic features, including its close interaction with the gut microbiota and pronounced hypoxic gradients. These characteristics likely confer strong tissue specificity on lactylation‐mediated regulatory networks. However, key CRC‐relevant aspects, such as the immunomodulatory effects of microbiota‐derived D‐lactate and the interaction between CRC‐specific oncogenic pathways (e.g., the APC/Wnt axis) and lactylation, have been largely overlooked in existing reviews.

Accordingly, after briefly summarising canonical lactylation mechanisms, this review focuses on CRC‐specific pathophysiological contexts. We examine how lactylation shapes the immunosuppressive TME, promotes liver pre‐metastatic niche formation, and contributes to resistance to 5‐fluorouracil and oxaliplatin. We also discuss pharmacological challenges that limit the clinical translation of lactylation‐targeted therapies in CRC. By integrating metabolism, epigenetics, and the TME, this review aims to provide a conceptual framework for precision diagnosis and treatment of CRC.

## 2 SOURCES, TRANSPORT AND FUNCTIONS OF LACTATE

Lactate, a hydroxycarboxylic acid, was first identified in 1780 by Carl Wilhelm Scheele. In humans, lactate is produced by cellular metabolism or obtained from external sources^13^ and exists as two stereoisomers, L‐lactate and D‐lactate.^14^ L‐lactate is the dominant form in mammalian cells, whereas D‐lactate is mainly generated by specific gut bacteria through fermentation.^15^ Under normoxic conditions, glucose‐derived pyruvate enters mitochondria and is fully oxidised to produce ATP. In contrast, under hypoxia, pyruvate is reduced to lactate by lactate dehydrogenase (LDH).^13^ Lactate production is also enhanced in pathological states such as ischaemia, infection and metabolic disorders, as well as during intense exercise.^16^

Because intracellular lactate accumulation inhibits glycolysis, cells must efficiently export lactate, whereas lactate‐consuming cells require active uptake. This process is mainly mediated by monocarboxylate transporters (MCTs), particularly MCT1 and MCT4. MCT1 is a high‐affinity transporter widely expressed in tumour cells and primarily mediates lactate uptake, but under hypoxic conditions, it can also facilitate lactate efflux.^17^ In contrast, MCT4 is a low‐affinity transporter specialised for lactate export and is transcriptionally upregulated by hypoxia‐inducible factor‐1α (HIF‐1α), thereby helping maintain intracellular pH homeostasis.^17^ Additionally, sodium‐coupled monocarboxylate transporters (SMCTs), such as SMCT1 and SMCT2, are crucial for lactate transport. SMCT1 possesses tumour‐suppressing functions and is often inactivated by hypermethylation in cancer.^18^ SMCT2 is highly expressed in CD4^+^ T cells and promotes lactate uptake, driving metabolic reprogramming and interleukin‐17 production, which supports immune cell retention at inflammatory sites.^19^, ^20^

Lactate was initially regarded as a metabolic waste product, but accumulating evidence has revealed its critical roles in diverse physiological and pathological processes. In the early 20th century, Warburg and colleagues showed that tumour cells preferentially convert glucose to lactate via glycolysis (the Embden–Meyerhof–Parnas pathway) even in the presence of oxygen, a phenomenon known as the Warburg effect, which supports tumour proliferation and invasion.^21^ Lactate serves as a significant energy substrate and is a primary fuel for the tricarboxylic acid cycle. It supports energy metabolism in the heart,^22^ skeletal muscles^23^ and brain tissue,^24^ and is also exploited by tumour cells.^25^ Moreover, lactate acts as a metabolic buffer linking glycolysis and oxidative phosphorylation (OXPHOS), thereby regulating intracellular redox homeostasis.^26^ It can also function as a carbon source in cancer cells by fueling amino acid and lipid metabolism, promoting fatty acid synthesis and suppressing lipolysis.^27^ When lactate accumulates in the TME, it acts as a signalling molecule that regulates cell proliferation, metabolism, angiogenesis, invasion and immune responses, thereby driving tumour initiation and progression.^28^, ^29^, ^30^, ^31^ In 2019, Zhao and coworkers^8^ reported that lactate can covalently modify lysine residues on histones to form lactylation (Kla), a novel PTM that directly regulates gene expression, thereby linking metabolic activity to epigenetic control.

Histone lactylation has been identified as a participant in various physiological and pathological processes, including inflammatory responses,^32^ wound healing,^33^ ischaemia‒reperfusion injury^34^ and remodelling of the TME.^35^ In addition to histones, lactylation also occurs on non‐histone proteins, modulating the activity of metabolic enzymes, transcription factors and signalling molecules,^36^ thereby influencing the progression of diseases such as prostate cancer, HCC, atherosclerosis and liver fibrosis.^37^, ^38^, ^39^, ^40^ Recently, Zhang et al.^41^ successfully distinguished three modification isomers: L‐lactylation of lysine (KL‐la), D‐lactylation of lysine (KD‐la) and N‐ε‐carboxyethyl‐lysine (Kce). Among them, KL‐la is the predominant and dynamically regulated form, closely linked to glycolytic activity and widely present across cell types.^41^ KD‐la suppresses immune activation and inflammatory signalling, thereby contributing to immune homeostasis.^42^

In CRC, enhanced glycolysis, widespread hypoxia and gut microbiota‐derived metabolites drive substantial lactate accumulation in the TME. This metabolic hallmark not only reshapes the tumour niche through microenvironmental acidification but also provides an abundant substrate reservoir for protein lactylation. Consequently, a systematic understanding of lactate production, transport and biological functions constitutes a fundamental prerequisite for elucidating how lactylation orchestrates epigenetic regulation, immune evasion and therapeutic resistance in CRC.

## 3 ADVANCEMENTS IN THE STUDY OF DYNAMIC REGULATORY MECHANISMS OF LACTYLATION

**FIGURE 1:**
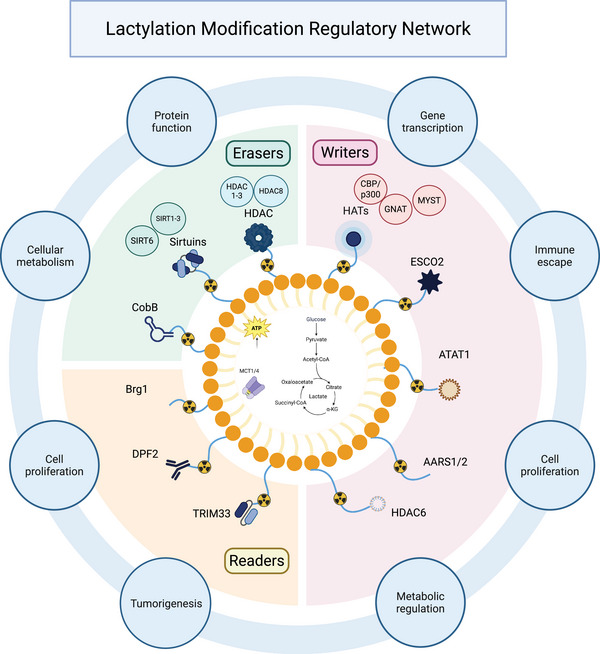
Schematic representation of the dynamic regulatory mechanisms of lactylation. Note: The diagram illustrates the metabolic origin of lactate, generated from glucose through glycolysis, and its transport into the nucleus or cytoplasm via monocarboxylate transporters (MCTs) (MCT1/4), where it contributes to lactylation. At the centre lies the lactylation regulatory network, organised in a radial hub composed of writers (e.g., HATs, ESCO2, ATAT1, AARS1/2, HDAC6), erasers (HDACs, Sirtuins, CobB) and readers (Brg1, DPF2, TRIM33). Peripheral annotations highlight the functional consequences of lactylation regulation, including altered cellular metabolism, proliferation, immune evasion and tumourigenesis, underscoring its pivotal role in colorectal cancer (CRC) progression.

The lactylation regulatory network comprises writers, erasers and readers, which together control the addition, removal and interpretation of lactylation signals. This system governs cellular metabolism, signalling and gene expression, and its dysregulation contributes to disease development. Understanding this dynamic network not only clarifies disease mechanisms but also provides new opportunities for diagnosis and therapy (Figure 1).

### 3.1 Lactylation writers

Lactylation writers catalyse the transfer of lactate‐derived groups, such as lactyl–coenzyme A (lactyl‐CoA), to lysine residues on target proteins. Identified writers fall into two main categories: classical acyltransferases and non‐canonical metabolic enzymes.

#### 3.1.1 Histone acetyltransferases

The histone acetyltransferase (HAT) family represents the best‐characterised class of lactylation writers and primarily uses lactyl‐CoA as a substrate. The CREB‐Binding Protein (CBP)/p300 family serves as the archetype; p300 was the first identified histone lactyltransferase.^8^ p300 catalyses lactylation on histones (e.g., H3K18la and H3K9la) and on non‐histone proteins such as HMGB1, α‐MHC and YY1, thereby promoting transcriptional activation.

Members of the GNAT family, including GCN5 (KAT2A), also exhibit lactyltransferase activity and can modify histones and immune checkpoint proteins, contributing to TME remodelling.^43^ In addition, MYST family enzymes such as KAT8 (MOF), HBO1 (KAT7) and KAT5 (Tip60) have been confirmed as lactylation writers. KAT8 acts as a broad lysine lactylation (Kla) writer, modifying translation elongation factors and metabolic enzymes, whereas HBO1 preferentially catalyses H3K9la.^44^

#### 3.1.2 Non‐canonical lactylation writers

Beyond classical HATs, several enzymes with unconventional catalytic activities have been identified as lactylation writers. Alanyl‐tRNA synthetases (AARS1/2) can generate lactyl‐AMP directly from lactate and ATP and catalyse lactylation of histones and non‐histone proteins, including p53 and cyclic GMP‒AMP synthase (cGAS), in a lactyl‐CoA‐independent manner.^45^

Notably, Histone Deacetylase 6 (HDAC6), traditionally considered a deacetylase, can switch to a lactyltransferase in high‐lactate conditions and modify cytoskeletal proteins such as α‐tubulin.^46^ In addition, ESCO2 and ATAT1 have been reported to regulate lactylation of DNA repair proteins and RNA‐modifying enzymes, respectively.^47^

#### 3.1.3 Non‐enzymatic lactylation mechanisms

In addition to enzymatic reactions, lactylation can also occur through non‐enzymatic pathways. Zhao et al. showed that during immune activation, downregulation of glyoxalase II (GLO2) leads to the accumulation of S‐D‐lactoylglutathione (SLG), which promotes D‐lactylation via a proximity‐transfer mechanism. In this process, a lactyl group is first attached to a cysteine residue and then transferred to a nearby lysine, enabling cytoplasmic protein lactylation independent of classical enzymes.

Together, lactyl‐CoA‐dependent reactions and alternative pathways mediated by lactyl‐AMP or SLG form the biochemical basis of lactylation. Importantly, aberrant expression of key writers such as p300 and KAT8 in CRC links metabolic dysregulation to epigenetic imbalance, highlighting these enzymes as potential targets for precision therapy.

### 3.2 Lactylation erasers

The dynamic balance of lactylation is maintained by delactylases or ‘erasers’, most of which belong to classical deacetylase families. Among zinc‐dependent histone deacetylases, HDAC1–3 function as major histone delactylases, with HDAC3 showing the strongest activity towards marks such as H3K18la and H4K5la; HDAC8 also exhibits delactylase activity.^48^ Similarly, the NAD^+^‐dependent Sirtuin family (SIRT1–3, SIRT6) possesses delactylase activity. SIRT2 is considered the most active cytoplasmic delactylase, whereas SIRT1 and SIRT3 primarily regulate the lactylation levels of metabolic enzymes, such as PKM2 and ENO1.^49^ In prokaryotes, Sir2‐like NAD‐dependent protein deacetylase in bacteria (CobB) functions as a bacterial delactylase, highlighting the evolutionary conservation of this regulatory mechanism.^50^ Dysregulation of these erasers disrupts lactylation homeostasis and can lead to aberrant oncogene activation, thereby promoting CRC progression.

### 3.3 Lactylation readers

Lactylation signals are interpreted by specific reader proteins that recognise Kla marks through specialised domains. Proteins containing YEATS (e.g., YEATS2) and PHD domains (e.g., DPF2 and TRIM33) bind lactylated lysines via hydrophobic pockets, thereby regulating chromatin remodelling and gene transcription.^51^ Although bromodomains typically recognise acetylated lysines, Brg1 can also bind H3K18la through its bromodomain, activating transcriptional programs associated with cellular plasticity.^52^

In summary, writers, erasers, and readers form a dynamic regulatory circuit that controls lactylation signalling. In CRC, dysregulated expression or activity of these regulators leads to aberrant lactylation of key substrates, thereby promoting malignant progression. The specific CRC‐related mechanisms are discussed in the following sections, and the major regulators and their therapeutic potential are summarised in Table 1.

**TABLE 1:** Key regulators of protein lactylation: integrated comparison of molecular structure, biological function, disease relevance and therapeutic targetability.

Category	Molecule	Functional role	Associated diseases/potential targets
Writer	p300 (histone acetyltransferase)^8^	Catalyses H3K18la modification, activates transcription, regulates immune evasion	Melanoma, Alzheimer's disease, glioblastoma; p300 inhibitor C646 shows targeting potential
	KAT8 (histone acetyltransferase)^10^	Catalyses K408 lactylation, involved in apoptosis regulation and skin remodelling	Colorectal cancer, ischaemia‒reperfusion injury, skin ageing; potential therapeutic inhibition target
	ESCO2 (non‐histone acetyltransferase)	Regulates virus‐related gene expression and lactylation	HSV‐1, KSHV, MPXV infections; potential viral regulation target
Eraser	SIRT1 (NAD+‐dependent deacetylase)^92^	Removes protein lactylation, modulates metabolism and cardioprotection	Heart failure, liver cancer; promising for immunotherapy combination
	HDAC3 (histone deacetylase)^93^	Removes H3/H4 lactylation, maintains chromatin homeostasis	Breast and pancreatic cancers; HDAC inhibitors show therapeutic potential
	SIRT6 (NAD+‐dependent deacetylase)	Regulates virus‐associated lactylation, involved in antiviral response	Virus‐associated tumours; potential new target for lactylation regulation
Reader	Brg1 (chromatin remodelling complex)^94^	Recognises H3K18 lactylation, promotes EMT and immune suppression	Liver and colorectal cancers; lactate‒Brg1 axis is a key pathway
	TRIM33 (transcriptional regulator)^51^	Recognises H3K18 lactylation, activates inflammatory pathways	Inflammatory tumour microenvironment; requires further validation
	DPF2 (histone reader protein)^95^	Recognises H3K14 lactylation, regulates cell cycle pathways	Cervical cancer, cell cycle dysregulation; early research stage

Abbreviation: EMT, epithelial‒mesenchymal transition.

## 4 LACTYLATION‐MEDIATED PROGRESSION OF CRC

Building on the metabolic features of lactate and the regulatory mechanisms of lactylation described above, increasing evidence indicates that lactylation plays a central role in CRC pathogenesis. By integrating metabolic reprogramming with epigenetic and immune signalling, lactylation promotes CRC cell proliferation, invasion, metastasis, immune evasion and resistance to chemotherapy (Table 2).

**TABLE 2:** Functional summary of lactylation targets.

Category	Target protein/gene	Modification site	Involved pathway/axis	Molecular mechanism	Therapeutic target type/effect
Proliferation	PFKP^96^	–	Glycolytic regulation axis	Lactylation reduces enzymatic activity, alters glycolysis, promotes CRC progression	Metabolic regulatory target
	β‐Catenin^59^	–	Wnt signalling pathway	Hypoxia‐induced lactylation activates Wnt signalling, facilitating CRC progression	Signalling pathway activator
	H3K18^54^, ^55^	H3K18la	H3K18la‒NSUN2‒m^5^C axis	Promotes NSUN2 transcription and m^5^C modification, activates autophagy, enhances proliferation	Epigenetic regulatory target
	RARγ^56^	–	H3K18la‒TRAF6/NF‐κB‒STAT3 axis	Lactate suppresses RARγ via H3K18la, activates STAT3, promotes tumourigenesis	Inflammatory pathway target
	CBX3^57^	K10la	CBX3‒H3K9me3 axis	Lactylation enhances H3K9me3 binding, regulates gene expression	Chromatin remodelling target
	eEF1A2^10^	K408la, etc.	KAT8‒eEF1A2 Kla axis	Enhances protein translation, promotes CRC development	Translational control target
Metastasis	LINC00152^66^	–	H3K18la‒YY1 axis	Lactylation reduces YY1 binding at promoter, enhances migration	lncRNA transcriptional regulation target
	GPR37^65^	–	GPR37‒Hippo‒CXCL1/5 axis	Activates Hippo pathway, upregulates H3K18la, promotes neutrophil recruitment and metastasis	Immune chemotaxis target
	RIG‐I (macrophages)^67^	–	Gut flora‐RIG‐I lactylation‒NF‐κB axis	Lactylation suppresses NF‐κB, promotes M2 polarisation and metastasis	Immune regulation target
Immune Escape	PCSK9^69^	–	PCSK9‒lactylation‒MIF axis	Lactylation upregulates MIF, promotes M2 macrophage polarisation, inhibits immunity	Tumour immune microenvironment target
	METTL3^70^	K281/K345la	H3K18la‒METTL3‒PD‐L1 axis	Lactylation enhances METTL3 expression/function, promotes immune evasion	Checkpoint co‐target
	PD‐L1^12^	–	PD‐L1 Kla stabilisation mechanism	Lactylation stabilises PD‐L1 protein, suppresses T‐cell activity	Immunotherapy resistance target
Drug resistance	MRE11^73^	K673la	MRE11‒HR repair axis	Enhances DNA binding and HR repair, induces chemoresistance	DDR pathway target
	NBS1^9^	K388la	NBS1‒HR repair axis	Enhances DNA repair capacity, increases resistance	DNA damage response target
	SMC4^75^, ^76^	–	SMC4‒lactate accumulation axis	Induces tumour cell dormancy, promotes chemoresistance	Cell cycle regulation target
	HDAC1^78^	K412la	HDAC1‒ferroptosis axis	Suppresses ferroptosis via lactylation, enhances resistance	Ferroptosis regulation target

Abbreviations: CRC, colorectal cancer; HR, homologous recombination; lncRNA, long non‐coding RNA; STAT3, signal transducer and activator of transcription 3.

### 4.1 Lactylation promotes malignant proliferation of CRC cells

**FIGURE 2:**
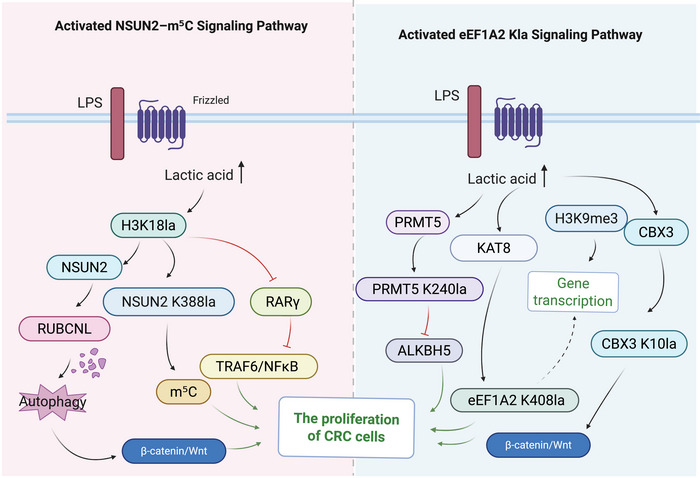
Molecular mechanisms by which lactylation promotes colorectal cancer (CRC) cell proliferation. Note: The diagram illustrates two major signalling pathways through which lactate enhances malignant proliferation in CRC cells. On the left, the NSUN2‒m^5^C axis: lactate induces H3K18la, upregulates NSUN2 expression and promotes NSUN2 K388la self‐lactylation, thereby driving m^5^C modification and autophagy activation, ultimately strengthening β‐catenin/Wnt signalling. On the right, the eEF1A2 Kla axis: lactate, via KAT8‐catalysed eEF1A2 K408la, engages regulatory interactions with PRMT5, ALKBH5 and CBX3 to facilitate protein translation and gene transcription. Together, these pathways converge to promote tumour cell proliferation, underscoring the central role of lactylation in CRC progression.

The mechanisms by which lactylation promotes the malignant proliferation of CRC cells are diverse, involving several key metabolic and epigenetic pathways, such as the NOP2/Sun RNA methyltransferase family member 2 (NSUN2)‒m^5^C circuit mediated by H3K18la or the KAT8‐catalysed eEF1A2 Kla axis that activates protein translation (Figure 2). TME acidification driven by metabolic reprogramming further supports these effects by enabling CRC cells to adapt to nutrient stress and sustain rapid growth. Recent studies have revealed that lactate contributes to the malignant behaviour of tumour cells through lactylation. For instance, Cheng et al.^53^ identified 637 lactylation sites on 444 proteins in SW480 colon cancer cells using liquid chromatography‒tandem mass spectrometry (LC‒MS/MS). Among these, lactylation of phosphofructokinase (PFKP K688la) reduced its enzymatic activity, providing negative feedback on glycolysis. This finding indicates that lactylation actively regulates CRC metabolism rather than representing a passive accumulation of metabolic byproducts.^53^

At the histone level, lactylation directly facilitates the malignant progression of CRC through transcriptional regulation. Chen et al.^54^ revealed that lactate accumulation in CRC cells not only activates the transcription of m^5^C methyltransferase (NSUN2) by promoting H3K18 lactylation but also induces lactylation at the K388 site of NOP2/Sun RNA Methyltransferase Family Member 2 (NSUN2 K388la), thereby accelerating CRC cell proliferation. Li et al.^55^ demonstrated that tumour‐derived lactate enhances H3K18la at the RUBCNL promoter, activating autophagy and promoting CRC cell survival. Under hypoxic conditions, inhibition of lactylation suppresses CRC cell growth and colony formation while inducing apoptosis, indicating that glycolysis‐driven lactate production fuels malignant proliferation. Furthermore, in the immune microenvironment, H3K18la inhibits retinoic acid receptor gamma (RARγ) expression in macrophages, weakening the TRAF6/NF‐κB axis function and thereby activating the signal transducer and activator of transcription 3 (STAT3) pathway in tumour cells, further promoting cancer cell expansion.^56^

Beyond histones, non‐histone lactylation plays a decisive role in translational regulation. Xie et al.^10^ elucidated the oncogenic mechanism of the MYST family member KAT8 (MOF) in CRC: KAT8 functions as a specific ‘writer’ catalysing the lactylation of eukaryotic translation elongation factor eEF1A2 at K408 (eEF1A2 K408la). This modification enhances the interaction between eEF1A2 and ribosomes, significantly increasing the rate of protein translation elongation, thereby meeting the immense demand for protein synthesis required for rapid CRC cell proliferation. This discovery not only reveals the ‘KAT8‒eEF1A2 Kla’ axis as a key driver of CRC proliferation but also explains the frequent overexpression of KAT8 in CRC.

Additionally, Duan et al.^57^ found that lactylation of Chromobox protein homolog 3 (CBX3) at K10 (CBX3 K10la) is significantly upregulated and regulates gene expression by enhancing the binding of CBX3 to H3K9me3, thereby promoting malignant proliferation and tumour growth in gastrointestinal cancers. Qu et al.^58^ reported that lactylation of PRMT5 at K240 (PRMT5 K240la) enhances the stability of SLC7A11 mRNA by inhibiting the expression of the N6‐methyladenosine (m^6^A) demethylase ALKBH5, thereby promoting ferroptosis resistance and driving cell proliferation and tumour progression in CRC. These studies indicate that non‐histone Kla modifications play a non‐negligible role in CRC, constituting an important supplement to lactylation‐promoted malignant behaviours.

At the level of canonical signalling pathways, the interplay between lactylation and the Wnt/β‐catenin pathway, a core driver of CRC, has garnered significant attention. Miao et al.^59^ found that hypoxic microenvironments significantly induce elevated β‐catenin expression and lactylation levels in CRC cells. Although the specific modification sites and enzymatic mechanisms remain to be fully elucidated, experiments confirmed that lactylated β‐catenin exhibits enhanced stability, leading to sustained activation of downstream Wnt target genes and driving CRC malignant proliferation. This finding directly couples metabolic stress (hypoxia/lactate) with the most classic oncogenic signal in CRC, providing a new perspective for understanding metabolically dependent proliferation in CRC.

Although AARS1/2 has mainly been reported to regulate p53 and YAP lactylation in gastric cancer,^45^ its frequent overexpression in metabolically active CRC suggests that similar non‐canonical lactylation of key tumour suppressors may also occur in CRC, representing an important direction for further investigation.

Furthermore, non‐histone lactylation mechanisms identified in HCC research provide important references for CRC. For example, lactylation of ATP‐binding cassette subfamily F member 1 (ABCF1) has been confirmed to translocate into the nucleus and activate the KDM3A‒HIF1A signalling axis, thereby promoting tumour growth^60^; meanwhile, ASH2L lactylation promotes angiogenesis through epigenetic regulation of vascular endothelial growth factor A (VEGFA) expression.^61^ These findings suggest that lactylation can directly act as a ‘switch’ for signal transduction. Given that Wnt/β‐catenin is the core oncogenic pathway in CRC, and lactylation of β‐catenin itself has been confirmed,^59^ it is reasonable to speculate that, similar to ABCF1/ASH2L in HCC, lactylation may directly modify core components of the Wnt pathway (such as APC or Axin), thereby becoming a direct driver of CRC malignant progression.

In summary, lactylation promotes CRC malignant proliferation through multiple levels, including metabolic regulation, histone transcriptional activation, non‐histone functional alteration and integration of key signalling pathways. This mechanism reveals the cross‐regulatory mode between metabolism and epigenetics and provides critical insights for identifying novel antitumour targets.

### 4.2 Lactylation promotes the invasion and metastasis of CRC cells

**FIGURE 3:**
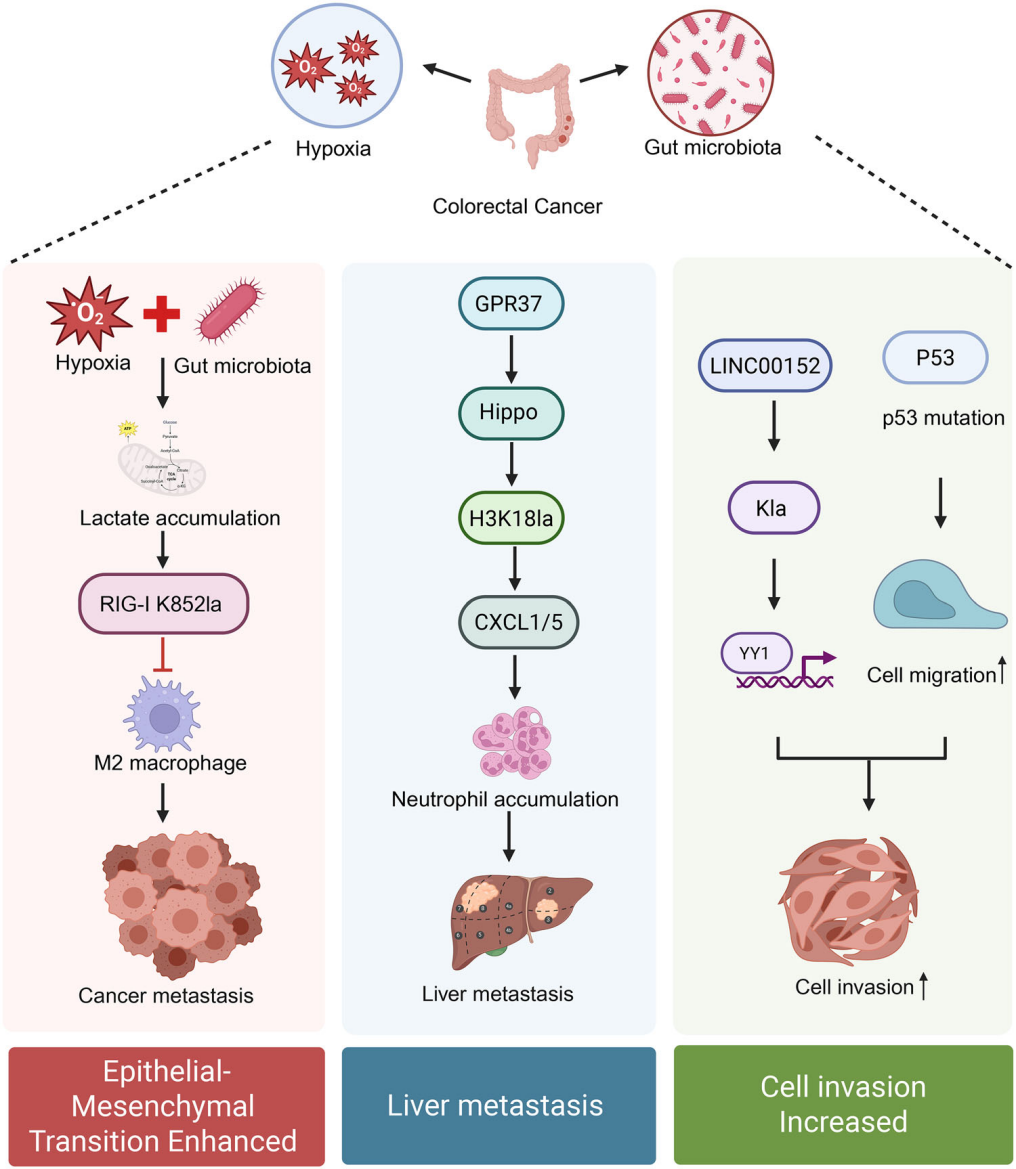
Mechanisms by which lactylation mediates colorectal cancer (CRC) cell migration and distant metastasis. Note: The upper section illustrates environmental factors such as hypoxia and gut microbiota that drive lactate accumulation in the tumour microenvironment (TME). On the left, lactate induces RIG‐I K852 lactylation, suppressing NF‐κB signalling and promoting M2 polarisation of tumour‐associated macrophages, thereby enhancing epithelial‒mesenchymal transition (EMT) and facilitating metastasis. In the middle, GPR37 activates the Hippo pathway and upregulates H3K18la, which transcriptionally elevates chemokine (C‒X‒C motif) ligand 1/5 (CXCL1/5), recruiting Neut and contributing to liver metastasis. On the right, lactylation of the LINC00152 promoter reduces its binding affinity with YY1 and enhances transcription, while p53 mutations (loss‐ or gain‐of‐function) further promote cellular migration and invasion. The bottom layer summarises the phenotypic outcomes: enhanced EMT, liver metastasis and increased cellular invasiveness. Kla denotes lysine lactylation.

Approximately 20%–25% of patients with CRC present with distant metastases, most commonly to the liver, at initial diagnosis, and nearly 50% of those with locally advanced disease eventually develop metastatic tumours. These statistics highlight the urgent need to elucidate the mechanisms that drive CRC metastasis. Distant metastasis in CRC is a multi‐step cascade involving epithelial‒mesenchymal transition (EMT) of cancer cells, extracellular matrix (ECM) remodelling, angiogenesis and the formation of the pre‐metastatic niche. As a metabolite‐derived epigenetic mark, histone Kla converts lactate signals into transcriptional programs that coordinate these events, thereby promoting CRC invasion and metastasis (Figure 3).

During the early stage of metastasis, lactylation acts as a key epigenetic driver of the EMT, which enables epithelial cells to acquire migratory and invasive properties. High lactate levels activate the transcription of EMT regulators (such as Snail, Slug and Twist) via histone H3K18la but also remodel chromatin structure through Brg1 (a lactylation reader) recognition of H3K18la. This enhances the transcriptional activity of cytoskeleton‐related genes, thereby endowing CRC cells with greater motility and invasiveness.^52^ Genetic alterations further amplify this process. In CRC, where TP53 is mutated in approximately 60% of cases, loss‐of‐function mutations enhance lactylation at the Snail promoter, accelerating EMT.^62^ Conversely, p53 gain‐of‐function mutations directly act on the promoter regions of CRC stem cell markers to promote tumour development.^63^ This interaction between mutation status and lactylation reveals a novel mechanism by which metabolism and genetic variation jointly drive EMT.

Angiogenesis is essential for CRC metastasis, and lactylation plays a central role in this process. Wang et al. showed that p300/CBP catalyses lactylation of YY1 at K183 (YY1 K183la), particularly under hypoxia. This modification enhances YY1 binding to the FGF2 promoter, increasing FGF2 expression and thereby promoting endothelial cell proliferation and tube formation.^64^ In CRC, this ‘p300‒YY1‒FGF2’ axis may directly explain the pathological phenomenon of increased microvessel density and elevated risk of hematogenous metastasis in lactate‐rich regions.

Regarding the metastatic microenvironment and ECM remodelling, lactylation promotes ‘seed and soil’ compatibility by regulating matrix degradation and immune cell recruitment. Lactate accumulation upregulates the expression of matrix metalloproteinases (MMPs), promoting collagen degradation and basement membrane disruption. Zhou et al.^65^ demonstrated that G protein‐coupled receptor 37 (GPR37) maintains high H3K18la levels by activating the Hippo pathway, subsequently inducing the secretion of CXCL1 and CXCL5. These chemokines recruit neutrophils to the liver, potentially constructing a pre‐metastatic niche through the formation of neutrophil extracellular traps (NETs), thereby assisting circulating tumour cells (CTCs) in liver colonisation.

Furthermore, non‐coding RNAs and the gut microbiota also participate in this regulatory network via lactylation. Long Intergenic Non‐Protein Coding RNA 152 (LINC00152), an oncogenic long non‐coding RNA (lncRNA), exhibits increased lactylation at its promoter region induced by Enterobacter lipopolysaccharide (LPS), which relieves transcriptional repression by inhibiting YY1 binding, thereby promoting CRC cell invasion.^66^ Simultaneously, Gu et al.^67^ found that the microbiota induces lactylation of retinoic acid‐inducible gene I (RIG‐I) protein at K852 via intratumoural glycolysis, inhibiting the NF‐κB signalling pathway and leading to M2 macrophage polarisation, thereby creating an immunosuppressive microenvironment that promotes liver metastasis.

In summary, these findings indicate that lactylation promotes CRC metastasis by coordinating EMT, angiogenesis, ECM remodelling, and immune suppression. Future studies should further explore how lactylation regulates integrin signalling and assess combination strategies targeting lactylation alongside anti‐angiogenic or immunotherapies.

### 4.3 Gut microbiota as a specific driver of the lactylation landscape in CRC

Unlike most solid tumours, CRC develops within the complex intestinal ecosystem, where the gut microbiota is a major component of the TME and a key source of lactate. Recent studies have identified a ‘microbiome–metabolism–epigenetics’ axis in which microbial dysbiosis reshapes the CRC lactylation landscape by altering local lactate availability.

#### 4.3.1 Bacterial‐derived lactate and D‐lactylation

The intestine harbours abundant lactate‐producing bacteria (e.g., *Lactobacillus*, *Bifidobacterium*). In CRC, dysbiosis often leads to an aberrant abundance of these lactate producers. Notably, bacteria produce not only L‐lactate but also significant amounts of D‐lactate. Due to the lack of efficient D‐lactate metabolising enzymes in humans, D‐lactate is prone to local accumulation in the gut. Zhao et al. demonstrated that microbial D‐lactate can be taken up by host cells and induce D‐lactylation of histone and non‐histone proteins through both non‐enzymatic and enzyme‐dependent pathways.^42^ This microbiota‐derived modification may exert functions distinct from L‐lactylation, particularly in regulating intestinal barrier integrity and mucosal immune responses.

#### 4.3.2 Microbiota metabolite‐driven immune microenvironment remodelling

Microbiota metabolites profoundly influence immune cell phenotypes through lactylation. Gu et al.^67^ confirmed that intratumoural bacteria produce large amounts of lactate via glycolysis, inducing lactylation of RIG‐I protein at K852 in macrophages. This modification suppresses the RIG‐I‐mediated antiviral signalling pathway and NF‐κB signalling, promoting macrophage polarisation towards the pro‐tumourigenic M2 phenotype, thereby creating an immunosuppressive microenvironment and facilitating CRC liver metastasis. Furthermore, colonisation by specific pathogens (e.g., *Fusobacterium nucleatum*) may indirectly enhance genome‐wide lactylation by upregulating host cell glycolysis, establishing a vicious ‘bacteria‒metabolism‒epigenetics’ cycle.

Collectively, the gut microbiota acts as an environmental ‘writer’ of lactylation in CRC. Defining the relationships between specific bacterial populations (e.g., D‐lactate producers) and site‐specific lactylation will open new avenues for microbiota‐based precision therapies. Although direct causal evidence in CRC patients remains limited, current data strongly support a CRC‐specific ‘microbiota–lactate–lactylation’ regulatory axis.

### 4.4 Lactylation promotes immune evasion in CRC

**FIGURE 4:**
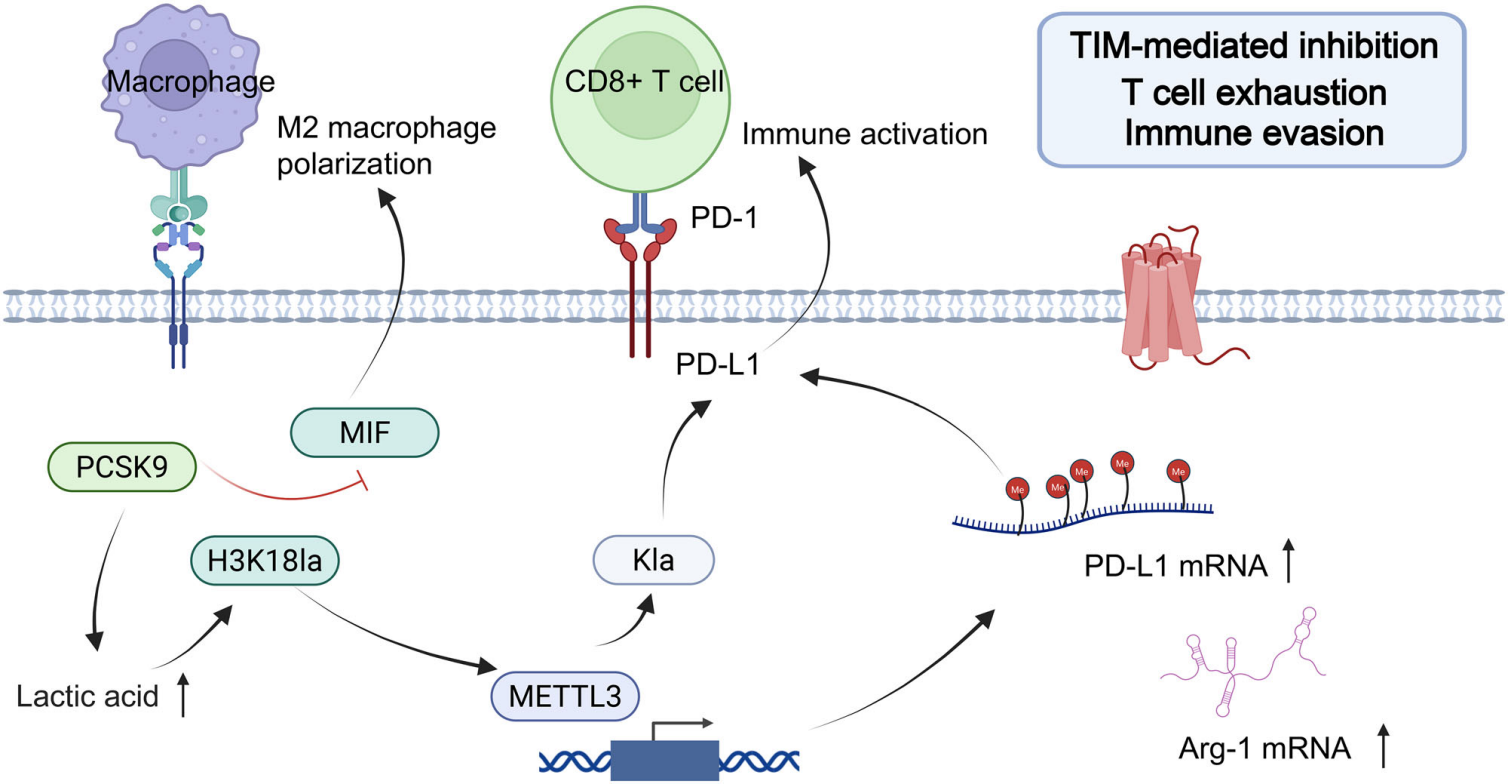
Mechanisms by which lactylation drives immune evasion in colorectal cancer (CRC). Note: Lactate accumulation triggers two major pathways: (1) H3K18la‒METTL3 axis: H3K18la upregulates METTL3 transcription, and lactylation of METTL3 itself enhances its N6‐methyladenosine (m^6^A) ‘writer’ activity. This activates the JAK1/signal transducer and activator of transcription 3 (STAT3) pathway and induces expression of PD‐L1 and arginase 1 (ARG1), leading to functional exhaustion of CD8‐positive T lymphocyte (CD8^+^ T cells). (2) PD‐L1 stabilisation: lactylation of PD‐L1 increases its stability, further suppressing T‐cell effector functions. Meanwhile, PCSK9 promotes upregulation of MIF, driving macrophage polarisation towards the M2 phenotype and forming tumour‐infiltrating immune cell (TIM)‐mediated immunosuppression, ultimately resulting in immune evasion.

Lactylation drives immune evasion in CRC by regulating METTL3, PD‐L1 and macrophage migration inhibitory factor (MIF), thereby linking metabolic reprogramming with immune epigenetic control (Figure 4). These mechanisms reshape the TME, promote infiltration of immunosuppressive cells, and enhance intrinsic immune escape of tumour cells.

In macrophage‐related pathways, Wang et al.^68^ identified that the key molecule in cholesterol metabolism, proprotein convertase subtilisin/kexin type 9 (PCSK9), is highly expressed in CRC. PCSK9 knockdown reduces histone lactylation and MIF expression, promotes antitumour M1 polarisation, and suppresses protumour M2 macrophages, thereby inhibiting CRC progression and metastasis. CRC models further show that PCSK9 elevates macrophage lactylation, and patients with high lactylation display stronger immune evasion and poorer responses to immunotherapy.^69^

Lactylation also plays a key regulatory role in tumour‐infiltrating immune cells (TIMs). A study by Wang and coworkers^70^ indicated that elevated expression of METTL3 in TIMs is associated with poor prognosis in colon cancer patients. Lactate in the TME upregulates METTL3 expression by inducing H3K18 lactylation, thereby promoting the immunosuppressive function of myeloid cells. This enzyme enhances immunosuppressive function through a dual mechanism: on one hand, it enhances Jak1 mRNA translation efficiency via m^6^A modification, activating the JAK1/STAT3 pathway to increase the expression of immunosuppressive molecules (PD‐L1 and Arg‐1). On the other hand, METTL3 itself is a direct substrate for lactylation; lactylation at K281 and K345 enhances its RNA‐binding ability and m^6^A methyltransferase activity. This dual mechanism collectively leads to enhanced immunosuppressive function of myeloid cells, promoting tumour immune evasion.

Intrinsic immune checkpoint regulation in tumour cells is directly controlled by lactylation writers. Tong et al.^12^ discovered that the GNAT family member GCN5 (KAT2A) is a key lactyltransferase for PD‐L1. GCN5 catalyses lactylation of the PD‐L1 protein, which hinders its lysosomal degradation pathway, thereby significantly increasing its stability and abundance on the tumour cell surface. This ‘GCN5‒PD‐L1 Kla’ axis not only enhances tumour cell resistance to T‐cell killing but also reveals the molecular mechanism by which a serine/glycine‐free diet affects immunotherapy efficacy through metabolic reprogramming.

In summary, lactylation promotes immune evasion in CRC by coordinating macrophage polarisation, microbial metabolism, tumour‐infiltrating immune cell function and immune checkpoint stabilisation. These mechanisms highlight lactylation as a central metabolic–epigenetic regulator and a promising target for overcoming immune resistance in CRC.

### 4.5 Lactylation remodelling in TME stromal cells

Beyond tumour and immune cells, stromal components within the TME, particularly cancer‐associated fibroblasts (CAFs) and vascular endothelial cells, play critical roles in CRC progression. Increasing evidence indicates that lactylation acts as a metabolic–epigenetic signal that drives phenotypic and functional remodelling of these stromal components.

#### 4.5.1 Metabolic conditioning of CAFs

CAFs represent the most abundant stromal cell population in the CRC microenvironment, promoting tumour progression through growth factor secretion and ECM remodelling. In a fibrosis model, Cui et al. showed that high lactate levels induce H3K18la in fibroblasts, activating profibrotic genes such as *Acta2*. A similar mechanism is likely operative in CRC: tumour‐derived lactate is taken up by quiescent fibroblasts and converted into lactylation signals, driving their transition into a pro‐tumourigenic myofibroblastic CAF (myCAF) phenotype. Activated CAFs further amplify this process by secreting additional lactate, forming a positive feedback loop. They also remodel the ECM, creating a physical barrier that limits drug penetration and immune cell infiltration, thereby facilitating CRC progression.

#### 4.5.2 Vascular endothelial cells and angiogenesis

Lactylation in vascular endothelial cells directly contributes to tumour angiogenesis. In hypoxic CRC, p300/CBP senses elevated lactate and catalyses YY1 K183la, which enhances FGF2 transcription and promotes endothelial cell proliferation, migration, and tube formation.^64^ Endothelial lactylation may also increase vascular permeability, facilitating the intravasation of CTCs and subsequent distant metastasis.

#### 4.5.3 Glial cells and the enteric nervous system

Although research has primarily focused on the central nervous system, Zu et al.^71^ found that SIRT2‐mediated delactylation plays a crucial role in glial cells. Given the dense enteric nervous system in the colon and rectum, lactate accumulation may alter enteric glial cells through lactylation, thereby promoting CRC progression and pain via perineural invasion.

In summary, lactylation functions as a metabolic language that coordinates signalling among tumour, stromal and neural cells in the TME. By reprogramming CAFs and endothelial cells, lactylation helps establish a malignant ecosystem that supports tumour growth and limits therapeutic efficacy.

### 4.6 Lactylation promotes chemotherapy resistance in CRC cells

**FIGURE 5:**
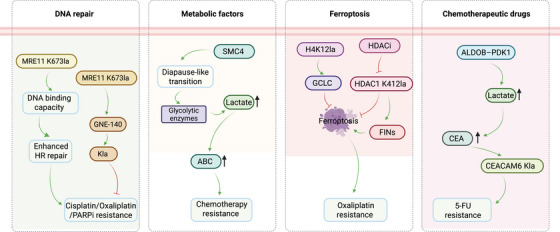
Mechanisms by which lactylation enhances chemoresistance in colorectal cancer (CRC) cells. Note: On the left, MRE11 K673la increases DNA‐binding capacity and enhances homologous recombination (HR), conferring resistance to cisplatin/oxaliplatin and PARP inhibitors (PARPi); inhibition of lactate dehydrogenase A (LDHA) with GNE‐140 reduces lactylation and attenuates resistance. In the mid‐left, sodium‐coupled monocarboxylate transporter 4 (SMC4) induces dormancy‐like transition and elevates glycolysis and lactate production, which in turn upregulates ATP‐binding cassette (ABC) transporters, promoting drug efflux and chemoresistance. In the mid‐right, H4K12la (catalysed synergistically by LDHA and p300) upregulates glutamate–cysteine ligase catalytic subunit (GCLC) to inhibit ferroptosis, while HDAC1 K412la promotes ferroptosis resistance; HDACi reduces lactylation at this site, increasing sensitivity to ferroptosis inducers (FINs). On the right, the ALDOB‒PDK1 axis elevates lactate and carcinoembryonic antigen (CEA) levels, enhancing CEACAM6 lactylation and protein stability, thereby driving resistance to 5‐fluorouracil (5‐FU).

Lactylation promotes chemoresistance in CRC by regulating DNA repair, metabolic reprogramming and ferroptosis (Figure 5). Lactylation of key proteins, including meiotic recombination 11 homologue A (MRE11), NBS1, HDAC1 and histone H4 lysine 12 lactylation (H4K12la), has been identified as a central mechanism underlying resistance to chemotherapy, immunotherapy and targeted therapy across cancers.^72^

In resistance driven by DNA damage repair, p300‐mediated modification plays a core role. He et al.^73^ demonstrated that p300/CBP catalyses lactylation of the key DNA repair protein MRE11 at K673. Highly lactylated MRE11 exhibits significantly enhanced binding affinity for DNA, thereby accelerating the homologous recombination (HR) repair process. This mechanism enables CRC cells to rapidly repair DNA damage induced by platinum‐based drugs or PARP inhibitors, leading to chemoresistance. Furthermore, Chen et al.^9^ found that targeting lactate dehydrogenase A (LDHA) to inhibit lactate production reduces NBS1 K388la levels, impairing MRN complex function and thereby overcoming this resistance. Utilising specific small peptides targeting MRE11 lactylation to inhibit CBP‐catalysed MRE11 K673 lactylation enhances HR deficiency, effectively improving the therapeutic efficacy of platinum drugs or PARP inhibitors against tumours.^74^

In CRC, metabolic reprogramming further drives lactylation‐dependent chemoresistance. Structural maintenance of chromosomes 4 (SMC4) induces a dormancy‐like state that upregulates glycolytic enzymes, increases lactate production and elevates lactylation, which in turn enhances ATP‐binding cassette (ABC) transporter expression and reduces sensitivity to chemotherapeutic agents.^75^, ^76^ Similarly, aldolase B (ALDOB) promotes the Warburg effect by activating pyruvate dehydrogenase kinase 1 (PDK1), increasing lactate production and carcinoembryonic antigen (CEA) expression. Lysine lactylation (Kla) stabilises CEA‐related cell adhesion molecule 6 (CEACAM6), thereby driving CRC cell proliferation and resistance to 5‐fluorouracil.^77^

Ferroptosis, an iron‐dependent form of regulated cell death, offers a promising strategy to overcome chemoresistance. Recent studies have uncovered a ‘metabolism–epigenetics–ferroptosis’ axis linking lactylation to ferroptotic control. Yang et al.^78^ showed that HDAC1 is functionally inactivated by auto‐lactylation at K412 (HDAC1 K412la) in CRC, a modification that is highly enriched in resistant tumours and suppresses ferroptosis. Importantly, HDAC inhibitors (HDACis) reduce HDAC1 K412la and restore CRC sensitivity to ferroptosis inducers (FINs).

Accumulating evidence supports a molecular link between lactate accumulation in the TME and ferroptosis regulation. Studies in HCC provide a useful model for CRC, showing that ABCF1 lactylation activates HIF‐1α signalling.^60^, ^61^ Because HIF‐1α directly induces ferritin heavy chain (FTH1) to sequester intracellular iron, this pathway can suppress lipid peroxidation and ferroptosis. Therefore, we propose the following novel chemoresistance mechanism: lactate accumulation in the TME induces lactylation of key proteins (such as ABCF1), which subsequently activates the HIF‐1α/FTH1 axis, reducing intracellular lipid peroxidation levels, and ultimately leading to CRC cell resistance to FINs and chemotherapeutic agents. Although this mechanism requires further direct validation in CRC, the aforementioned HCC studies provide a highly credible theoretical framework for understanding lactylation‐mediated ferroptosis evasion in CRC.

Subsequently, Deng et al. discovered^79^ that colorectal cancer stem cells (CCSCs) acquire chemoresistance through H4K12 lactylation (H4K12la)‐mediated metabolic reprogramming. LDHA and p300 cooperatively generate H4K12la, which upregulates glutamate–cysteine ligase catalytic subunit (GCLC), suppresses ferroptosis and confers resistance to chemotherapeutic agents such as oxaliplatin.

Overall, lactylation exerts broad effects on chemoresistance by coordinating DNA repair and metabolic pathways. Targeting lactylation of key regulators, including LDHA and MRE11, offers a promising strategy to restore drug sensitivity and improve chemotherapy efficacy in CRC.

### 4.7 Validation of target specificity via gene knockout models

Although small‐molecule inhibitors are widely used in cancer research, their off‐target effects can confound interpretation. To define the specific roles of lactylation in CRC and exclude nonspecific metabolic effects, recent high‐impact studies have applied gene knockout (KO) and site‐directed mutagenesis approaches. These genetic strategies provide gold‐standard evidence for the causal and target‐specific functions of lactylation.

First, regarding the specific correspondence between metabolic enzymes and modification substrates, Chen et al.^9^ utilised CRISPR/Cas9 technology to generate *LDHA*‐KO CRC cell lines. Experiments confirmed that *LDHA* deletion specifically blocked intracellular lactate production, leading to the near‐complete abolition of NBS1 K388la modification levels. Furthermore, re‐supplementation with exogenous lactate specifically restored this modification and the cells' HR repair capacity, thereby genetically locking the specificity of the ‘LDHA‒lactate‒NBS1 lactylation’ axis.

Second, concerning the functional specificity of writer enzymes, Xie et al.^10^ generated an intestine‐specific *Kat8* conditional KO mouse model (*Vil‐Cre; Kat8 fl/fl*). In vivo deletion of *Kat8* completely abolished eEF1A2 K408 lactylation (K408la), which could not be compensated by other acetyltransferases and led to marked suppression of CRC tumour growth. This result confirms KAT8 as the site‐specific lactyltransferase for eEF1A2.

Additionally, regarding the validation of substrate protein modification sites, the use of gene editing to construct lysine‐to‐arginine (K‐to‐R) mutants provides the most direct evidence. For instance, Wang et al.^70^ mutated the K281 and K345 sites of METTL3 to arginine (METTL3‐2KR). Although the mutant retained protein expression, it completely lost lactylation and its ability to promote m^6^A modification and tumour immune evasion. Together, KO and point‐mutation studies demonstrate that lactylation is not a metabolic byproduct but a specific and functionally essential modification driving CRC progression.

### 4.8 Functional controversies and context dependency of lactylation in CRC

Although most evidence supports a pro‐tumourigenic role for lactylation in CRC, its functions are highly context‐dependent and can be double‐edged.

In the immune microenvironment, lactylation shows strong cell type and context specificity. In CRC, histone lactylation in macrophages promotes M2 polarisation and immunosuppression.^70^ In contrast, in autoimmune and acute inflammatory settings, lactylation suppresses pro‐inflammatory gene expression and exerts protective effects.^8^ These opposing outcomes likely reflect differences in metabolic substrates and signalling pathways between chronic tumour inflammation and acute immune responses.

Controversy also exists regarding the role of lactylation in tumour cells. Although most studies emphasise its pro‐proliferative and pro‐metastatic effects, excessive lactate and lactylation can induce acidosis, leading to cell‐cycle arrest or apoptosis. In some non‐CRC models, site‐specific lactylation stabilises or activates tumour suppressors such as wild‐type p53, thereby restraining tumour growth.^80^ These findings highlight the context‐dependent and substrate‐specific nature of lactylation, indicating that its net effect depends on whether modified proteins function within oncogenic or tumour‐suppressive pathways.

Furthermore, lactylation functions vary by subcellular localisation. Nuclear histone lactylation primarily regulates gene transcription and typically promotes malignant phenotypes, whereas cytoplasmic lactylation of metabolic enzymes (e.g., PKM2, PGK1) is more involved in the fine tuning of metabolic flux. Studies have found that in CRC cells, lactylation of phosphofructokinase (PFKP) at K688 directly inhibits its enzymatic activity, forming a negative feedback loop for glycolysis.^53^ This suggests that in early‐stage CRC, cytoplasmic lactylation might temporarily limit tumour growth by inhibiting glycolysis, whereas as the disease progresses, nuclear epigenetic regulation gradually dominates, shifting towards a pro‐tumourigenic mode.

In summary, lactylation is not inherently pro‐tumourigenic; its effects depend on cell type, microenvironmental acidity, disease stage and substrate specificity (Table 3). Future studies should resolve the spatiotemporal dynamics of lactylation at single‐cell resolution to avoid unintended effects from nonselective therapeutic inhibition.

**TABLE 3:** Summary of context‐dependent and conflicting roles of lactylation in tumour biology.

Context/cell type	Target/substrate	Observed effect	Biological outcome	Potential mechanism/reason	References (PMID)
CRC/tumour Cells	Histone H3K18la	Pro‐tumour	Promotes EMT, proliferation, and metastasis	Activates oncogenic transcription programs (e.g., YAP, Snail) through chromatin remodelling	PMID: 35320754; PMID: 38769664; PMID: 37615625^54^, ^55^, ^70^
Macrophages (M2)	Histone H3K18la/METTL3	Pro‐tumour	Immunosuppression	Induces Arg1 and VEGF expression; enhances m^6^A modification for STAT3 activation	PMID: 35320754^70^
Macrophages (acute inflammation)	Histones (H3K18la)	Anti‐inflammatory/homeostatic	Resolution of inflammation/wound healing	Activates ‘lactate clock’ genes to restore homeostasis after bacterial challenge	PMID: 31645732^8^
Metabolic stress/CRC cells	PFKP (metabolic enzyme)	Antitumour (negative feedback)	Inhibition of glycolysis	PFKP‐K688la attenuates enzyme activity to prevent excessive lactate accumulation	PMID: 38155775^53^
Sepsis/endothelium	HMGB1	Pro‐inflammatory	Increased vascular permeability	p300‐mediated lactylation promotes HMGB1 exosome secretion	PMID: 34363018^83^

Abbreviations: CRC, colorectal cancer; EMT, epithelial‒mesenchymal transition; m^6^A, N6‐methyladenosine; STAT3, signal transducer and activator of transcription 3; VEGF, vascular endothelial growth factor.

## 5 CLINICAL PROSPECTS OF TARGETING LACTYLATION FOR CRC THERAPY

**FIGURE 6:**
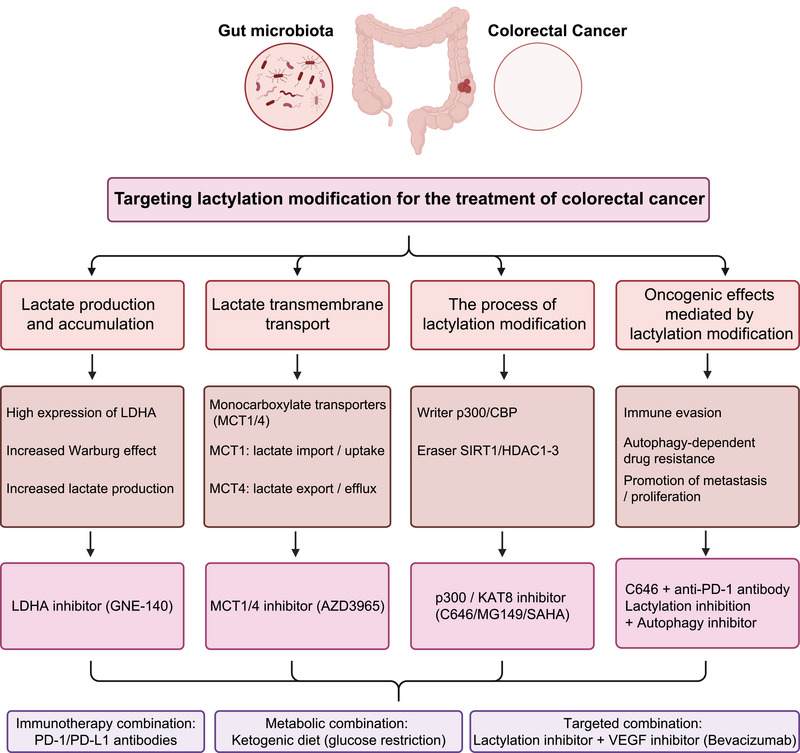
Strategic pathways for targeting lactylation in colorectal cancer (CRC) therapy. Note: The diagram outlines therapeutic strategies from left to right. (1) Targeting lactate production and accumulation, including the Warburg effect and lactate dehydrogenase A (LDHA) overexpression, with inhibitors such as GNE‐140; (2) blocking lactate transmembrane transport via monocarboxylate transporter (MCT) 1/4 inhibition (e.g., AZD3965); (3) regulating the lactylation process by inhibiting writers such as p300/CBP and KAT8 (with agents including C646 and MG149) or modulating delactylases/deacetylases (e.g., suberoylanilide hydroxamic acid [vorinostat]; SAHA); (4) interfering with oncogenic outcomes mediated by lactylation, including immune evasion, autophagy‐dependent resistance, and tumour invasion/proliferation; and (5) combination therapies, such as immune checkpoint inhibitors (anti‐PD‐1/PD‐L1 antibodies), metabolic interventions (ketogenic/low‐glucose diets), and synergistic regimens with anti‐vascular endothelial growth factor (VEGF) agents (e.g., bevacizumab) or autophagy inhibitors.

With the deepening elucidation of CRC metastasis mechanisms, targeting lactylation has emerged as a promising strategy to overcome chemoresistance and improve immunotherapy. Given its broad regulatory roles in CRC, current therapeutic efforts focus on inhibiting lactate production or transport, modulating lactylation enzymes and developing rational combination therapies (Figure 6).

### 5.1 Potential therapeutic strategies and drug development progress

Effective precision therapy requires accurate patient stratification. Because of CRC heterogeneity, only a subset of patients is likely to benefit from lactylation‐targeted approaches. Thus, lactylation‐based biomarkers are essential. For example, H3K18la levels in tumour tissues can serve as a direct indicator of lactylation activity, while plasma lactate or expression of metabolic enzymes (e.g., LDHA) may provide complementary markers. Integrating these biomarkers with genetic features, such as TP53 status, will be critical for matching the right therapy to the right patient in future clinical trials.

#### 5.1.1 Blocking the source and transport of lactate metabolism

Inhibiting lactate generation and transport is a direct strategy to suppress lactylation. LDHA inhibitors, such as GNE‐140, reduce lactate production and thereby lower global lactylation levels. To block lactate transport, the MCT1 inhibitor AZD3965 has entered phase I/II clinical trials, aiming to disrupt tumour metabolic coupling and improve the immune microenvironment.^81^ In addition, transcriptomic metabolic subtyping indicates that combining a ketogenic diet with LDHA inhibition can further restrict glucose availability and may be particularly effective in TP53‐mutant CRC.^82^

#### 5.1.2 Modulating the activity of lactylation enzymes

Targeting lactylation ‘writers’ and ‘erasers’ is an active area of therapeutic development, although clinical translation remains early. p300/CBP inhibitors, such as CCS1477, have entered clinical trials for hematologic malignancies (NCT03568126) and are under preclinical evaluation in CRC. The p300/CBP inhibitor C646 reduces lactylation of key proteins, including HMGB1, and shows strong synergy with the BRAF inhibitor vemurafenib in CRC models.^73^, ^83^ Similarly, the KAT8 inhibitor MG149 suppresses CRC growth by blocking eEF1A2 lactylation‐dependent translation.

Regarding erasers, although HDACis (e.g., Vorinostat, Entinostat) have been tested in CRC (e.g., NCT03986064), these trials targeted acetylation. As HDAC1–3 also act as delactylases, reanalysis of these data may reveal lactylation‐dependent effects. Efforts are now focused on developing selective lactylation modulators to avoid the off‐target toxicity of conventional HDACis, consistent with the view that precise control of writers and erasers is key to restoring therapeutic sensitivity.^84^

#### 5.1.3 Combinatorial immunotherapy strategies

The tight link between lactylation and immune evasion supports combination therapies. For example, PD‐1 blockade combined with LDH inhibition markedly enhances antitumour activity.^85^ In addition, targeting H3K18la‐driven RUBCNL reverses hypoxia‐induced resistance to bevacizumab.^55^ Together, these findings indicate that combining lactylation modulators with immune checkpoint inhibitors or anti‐angiogenic agents represents a promising direction for clinical translation. Key targets and strategies are summarised in Table 4.

**TABLE 4:** Summary of therapeutic strategies targeting lactylation in colorectal cancer (CRC).

Drug name	Target	Mechanism of action	Research stage	Challenges/limitations
GNE‐140^97^	LDHA	Inhibits lactate production, reduces H3K18la formation, reverses autophagy‐mediated resistance	Animal study	Systemic toxicity due to inhibition of glycolysis in normal tissues (e.g., RBCs).
AZD3965^98^	MCT1	Blocks lactate efflux, alleviates immunosuppressive TME	Clinical phase I/II	Compensation by MCT4 upregulation; potential cardiac toxicity.
C646^99^	p300	Reduces histone lactylation, reverses PD‐L1 expression	In vitro/animal study	Poor selectivity against acetylation; low bioavailability.
MG149^10^	KAT8	Suppresses eEF1A2 lactylation, reduces protein translation rate	In vitro	Off‐target effects on histone acetylation; lack of in vivo stability data.
SAHA (Vorinostat)^100^	HDAC	HDAC inhibition, affects ferroptosis‐related Kla sites	Clinically approved (for other cancers)	Non‐specific; affects multiple post‐translational modifications (acetylation/crotonylation).
Resveratrol^101^	SIRT1/3	Activates delactylation function, enhances immune clearance	Preclinical	Low bioavailability; rapid metabolism; pleiotropic effects.
β‐Alanine^102^	AARS1	Disrupts interaction between writer and lactate, inhibits lactylation	In vitro study	High concentration required; potential interference with protein synthesis.
Combination: CPI‐1612 + anti‐PD‐1 antibody^103^	EP300/CBP + PD‐1/PD‐1 pathway	Disrupts the ‘lactylation–immunosuppression’ loop, enhances T‐cell function and antitumour efficacy	Animal study	Risk of immune‐related adverse events; optimal dosing schedule unclear.
Combination: ketogenic diet + LDHA inhibition^104^	Glycolysis + lactate metabolism	Limits glucose supply + lactate generation, suppresses TME formation	Preclinical	Patient compliance issues; risk of weight loss/cachexia in advanced CRC.

Abbreviations: LDHA, lactate dehydrogenase A; MCT, monocarboxylate transporter; TME, tumour microenvironment.

### 5.2 Pharmacological challenges and limitations in developing lactylation‐targeted drugs

As summarised in Table 3, although strategies targeting lactate metabolism and lactylation show promise in preclinical models, their clinical translation is limited by poor selectivity, toxicity and low bioavailability.

Despite promising results in preclinical CRC models, translating strategies that target lactate metabolism and lactylation into the clinic remains challenging. Most current approaches rely on drug repurposing, which limits selectivity, increases toxicity and compromises bioavailability.^86^

Limited specificity and off‐target effects are major barriers. Most available lactylation modulators are broad‐spectrum enzyme inhibitors. For example, HDACis such as SAHA increase lactylation but primarily block deacetylation, causing global acetylation imbalance and cytotoxicity. Likewise, the KAT8 inhibitor MG149 was developed as a lysine acetyltransferase inhibitor and has not been shown to selectively inhibit lactylation without affecting acetylation.^10^, ^87^ Because lactylation and acetylation share many writers and erasers, developing isoform‐specific or allosteric inhibitors that selectively target lactylation remains a key unmet need.

Metabolic compensation and bioavailability further complicate therapy. Although LDHA inhibitors (e.g., GNE‐140) effectively reduce lactate in vitro, systemic inhibition can cause haemolytic anaemia and muscle toxicity, as normal tissues also depend on glycolysis.^88^ In addition, CRC cells can bypass glycolytic blockade by using alternative fuels such as fatty acids or glutamine, leading to rapid drug resistance.^89^

Research on lactate stereoisomer specificity remains limited. Both L‐lactate and microbiota‐derived D‐lactate are present in humans,^90^ yet most studies and drug development efforts focus almost exclusively on the L‐lactate pathway. Little is known about the distribution, functional targets or druggability of D‐lactate‐derived lactylation, despite its distinct role in immune regulation.^42^ Neglecting this isomer may compromise therapeutic predictability.

Therefore, future drug development should prioritise proteolysis‐targeting chimera (PROTAC)‐based degradation of specific lactylation enzymes and small‐molecule inhibitors of reader proteins (such as Brg1) to achieve more precise intervention.

### 5.3 Next‐generation therapeutic paradigm: precision intervention targeting specific lactylation sites

Given the limitations of broad‐spectrum enzyme inhibitors, precise targeting of oncogenic lactylation sites represents a key future direction. Recent advances in HCC provide a proof of concept: an intelligent nanocomposite (mPEG‐NH_2_/2‐FPBA/TubA) was developed to selectively inhibit ABCF1 K430 lactylation, thereby suppressing tumour growth without disrupting lactylation on other proteins.^91^

This ‘site‐specific intervention’ concept is particularly important for CRC. For example, targeting the aforementioned CRC‐specific oncogenic modification—eEF1A2 K408la—future efforts could develop similar nanodelivery systems or peptide‒drug conjugates to precisely block modification at this site without interfering with KAT8's acetylation function on other substrates. This paradigm shift from ‘pan‐enzyme inhibition’ to ‘site‐precise targeting’ will significantly reduce off‐target toxicity and is the ultimate goal for achieving precision epigenetic therapy in CRC.

## 6 CONCLUSION AND PERSPECTIVES

Lactylation plays a central role in CRC initiation and progression. Metabolically, it reprograms intracellular pathways to sustain energy production and biosynthesis, supporting rapid tumour growth. During metastasis, lactylation regulates signalling networks that control cell adhesion and migration, thereby facilitating invasion. It also promotes immune evasion by shaping an immunosuppressive TME and contributes to chemotherapy resistance by regulating therapy‐related proteins and pathways.

**FIGURE 7:**
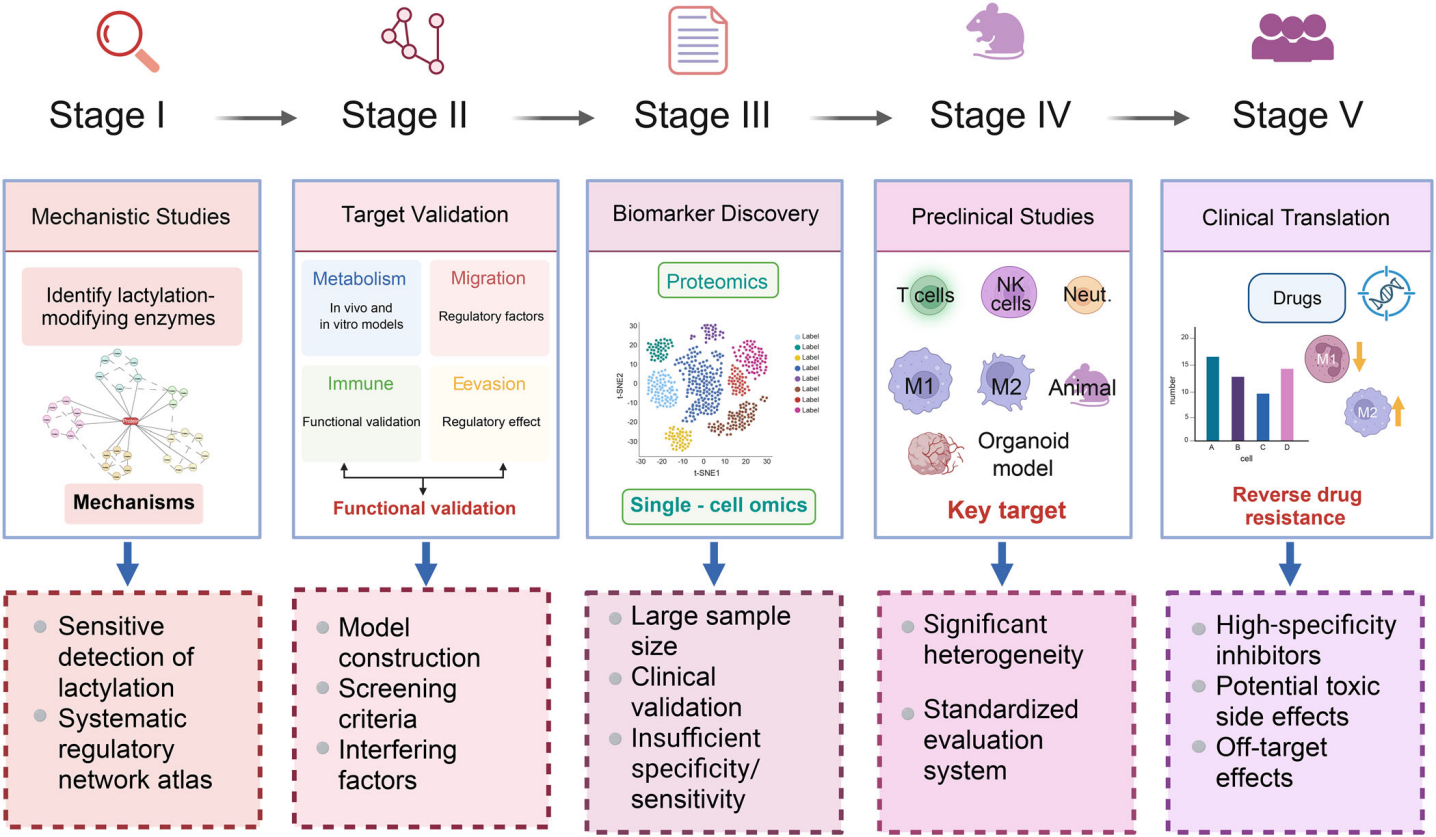
Integrated schematic of the mechanistic logic of lactylation‐mediated colorectal cancer (CRC) development from metabolic origin to therapeutic targeting. Note: The diagram adopts a radial layout, with the lactylation regulatory network (writers, erasers and readers) at the centre. Four major functional modules radiate outward, illustrating how lactylation drives malignant proliferation, promotes invasion and metastasis, mediates immune evasion and induces chemoresistance. The right panel depicts potential therapeutic interventions, including enzyme inhibitors, transporter inhibitors, combinational strategies and emerging technological platforms. The lower section summarises current research challenges and future directions. Collectively, the figure presents an integrated overview of lactylation, spanning from its metabolic origin to therapeutic targeting in CRC.

Despite these advances, the study of lactylation in CRC remains in its early stages. Although key enzymes such as p300 and GCN5 have been identified, the full regulatory network and its interactions with other PTMs, including phosphorylation and acetylation, require further investigation. Clinically, site‐specific marks such as H3K18la show promise as biomarkers, but their diagnostic and prognostic value must be validated in large patient cohorts. Developing highly selective and low‐toxicity agents that target lactylation‐related pathways will be essential for translating these discoveries into effective CRC therapies (Figure 7).

The relative emphasis on histone versus non‐histone lactylation also warrants reassessment. Although early studies focused on nuclear H3K18la‐mediated transcription, increasing evidence indicates that cytoplasmic non‐histone lactylation plays an equally important, and often more direct, role in CRC progression. For example, KAT8‐mediated eEF1A2 lactylation regulates protein synthesis, while HDAC6‐mediated lactylation of cytoskeletal proteins promotes cell migration. However, the lactylation landscape of cytoplasmic proteins, particularly those involved in EMT and cytoskeletal remodelling, remains poorly defined. High‐resolution proteomics will be essential to map this network and clarify its role in CRC metastasis.

Major knowledge gaps remain. A key priority is to identify lactylation‐specific readers that mediate signal transduction. Although YEATS and PHD domains can recognise lactylated lysines, no universal lactylation‐binding module equivalent to the bromodomain has been defined. Developing photo‐crosslinking and chemical probe–based proteomic tools to capture CRC‐specific readers will be essential for mapping lactylation signalling and identifying therapeutic targets.

A second major challenge is to define the crosstalk between lactylation and other PTMs. Lactylation interacts with acetylation, methylation and phosphorylation to form complex regulatory networks. In HCC, ASH2L lactylation recruits the MLL complex, enhances H3K4me3, and cooperatively activates VEGFA transcription,^61^ indicating that lactylation can act upstream of epigenetic control. In CRC, key drivers such as β‐catenin and p53 are tightly regulated by phosphorylation and acetylation. Whether lactylation competes with acetylation for lysine residues or alters protein conformation to influence kinase binding remains a critical question for defining the regulatory landscape of CRC.

**FIGURE 8:**
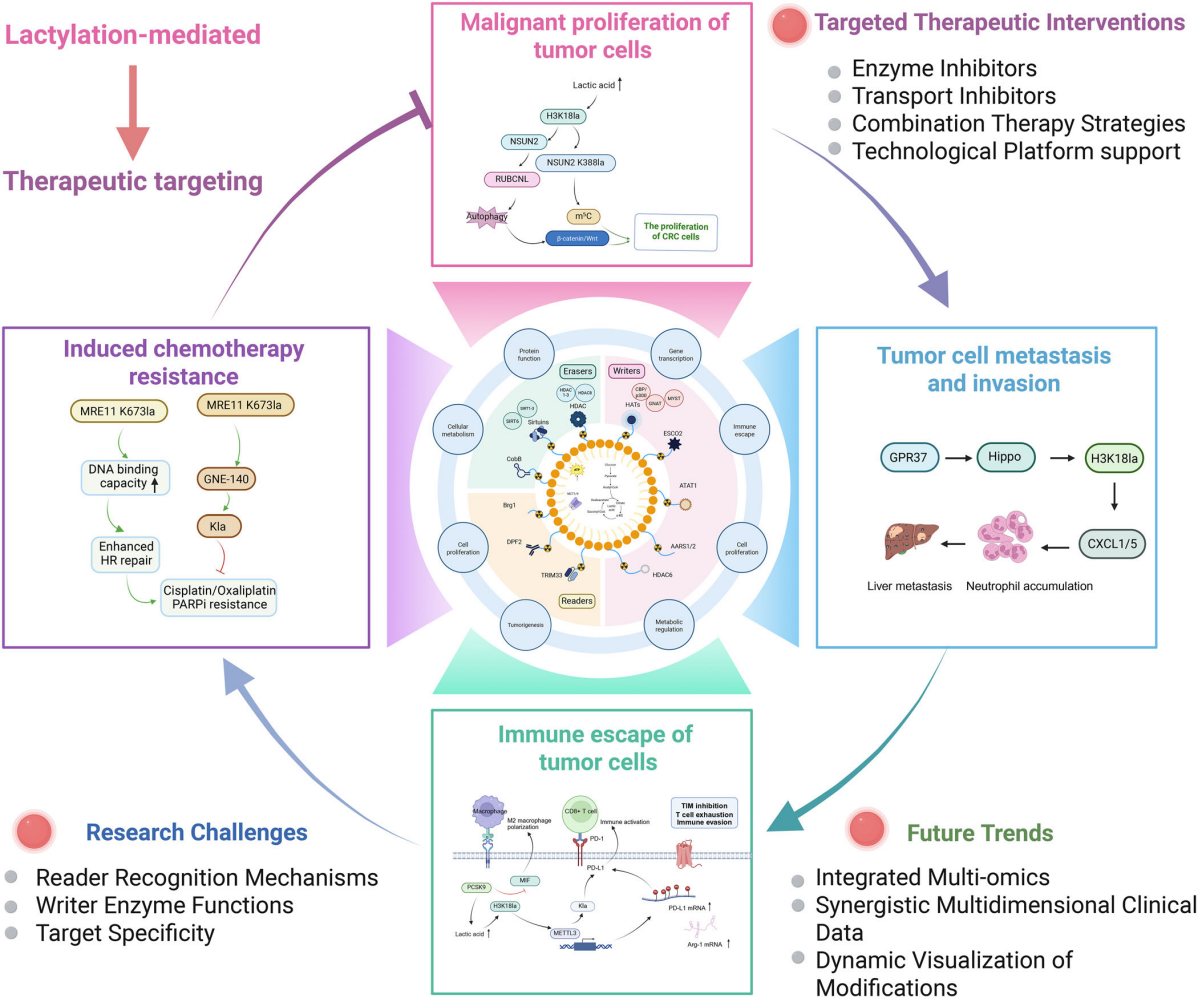
Roadmap for future research on lactylation in colorectal cancer (CRC). Note: The roadmap is organised into five stages. Stage I (mechanistic studies): identification of lactylation enzymes and substrates, characterisation of regulatory crosstalk, and construction of interaction networks. Stage II (target validation): validation of key factors in vitro and in vivo for their causal roles in metabolism, migration and immune evasion. Stage III (biomarker discovery): integration of proteomics and single‐cell omics to identify and clinically validate translational biomarkers. Stage IV (preclinical studies): evaluation of critical targets and candidate drugs in mouse and organoid models. Stage V (clinical translation): assessment of safety and efficacy of therapeutic agents and combination strategies, with exploration of their potential to overcome drug resistance. Key challenges are noted below each stage, including detection sensitivity and standardisation, model heterogeneity, specificity and sensitivity with multicentre validation, assessment systems, and issues of inhibitor specificity, off‐target effects, and toxicity. NK cell, natural killer cell.

With advances in high‐resolution proteomics and gene‐editing technologies, therapies targeting lactylation are increasingly feasible. Lactylation‐directed agents have already shown efficacy in CRC models, with the potential to overcome resistance and improve clinical outcomes. Integrating lactylation profiles with genetic and clinical parameters will enable more accurate patient stratification and personalised treatment. Nonetheless, further basic and clinical studies are required to validate these approaches and translate them into effective therapies (Figure 8).

## AUTHOR CONTRIBUTIONS

*Conceptualisation and writing—original draft*: Ming Liu. *Validation and formal analysis*: Weiwei Li. *Data curation and visualisation*: Yi Ji. *Supervision*: Yanqing Chen. *Project administration*: Guoli Wei. *Investigation*: Jiege Huo. *Writing—review and editing*: Tao Gui. All the authors reviewed and approved the final version of the manuscript.

## CONFLICT OF INTEREST STATEMENT

The authors declare they have no conflicts of interest.

## ETHICS STATEMENT

Not applicable.

## Data Availability

All data generated or analysed during this study are included in this article and/or its Supporting Information. Further enquiries can be directed to the corresponding author.
